# Determinants of Vaginal Microbiota Composition

**DOI:** 10.3389/fcimb.2020.00467

**Published:** 2020-09-02

**Authors:** Yumna Moosa, Douglas Kwon, Tulio de Oliveira, Emily B. Wong

**Affiliations:** ^1^Africa Health Research Institute, Durban, South Africa; ^2^KwaZulu-Natal Research and Innovation Sequencing Platform, University of KwaZulu Natal, Durban, South Africa; ^3^Ragon Institute of Massachusetts General Hospital, Massachusetts Institute of Technology and Harvard University, Cambridge, MA, United States; ^4^Division of Infectious Diseases, Massachusetts General Hospital, Boston, MA, United States; ^5^Harvard Medical School, Boston, MA, United States; ^6^Centre for the AIDS Programme of Research in South Africa, Durban, South Africa; ^7^Division of Infection and Immunity, University College London, London, United Kingdom

**Keywords:** vaginal microbiota, vaginal dysbiosis, genital inflammation, microbiota transmission, HIV acquisition

## Abstract

There is increasing evidence that the composition of a woman's vaginal microbiota significantly influences her sexual and reproductive health, including her risk of miscarriage, preterm birth, HIV and other sexually transmitted infections. Efforts to modulate the vaginal microbiota using antibiotic or probiotic therapy have shown limited lasting or reliable success. To explore the natural dynamics and causal pathways responsible for heterogeneity of vaginal microbiota composition we review the existing literature on its determinants, from the perspective of microorganism- and host-related factors. We then discuss how molecular approaches can be harnessed to advance our understanding of individual and population-level vaginal microbiota composition patterns. Work has been done to investigate determinants of microbial composition patterns in other body niches, but very little in the female genital tract so far. There is an urgent need to better understand vaginal microbiota composition patterns, across the lifespan, outside of the context of sexual health clinics, and in Sub-Saharan African women in whom vaginal microbiota composition may be a risk factor for HIV acquisition. More work is needed to clarify causal relationships between clinical symptoms, host genetic, host behavior, and molecular vaginal microbiota profiles. These insights will lay the groundwork for novel and targeted interventional approaches to improve women's sexual and reproductive health.

## Introduction

There is increasing evidence that the composition of a woman's vaginal microbiota significantly influences her sexual and reproductive health, including her risk of adverse birth outcomes including miscarriage and preterm birth and acquisition of infections including HIV and other sexually transmitted pathogens.

**Figure 1:**
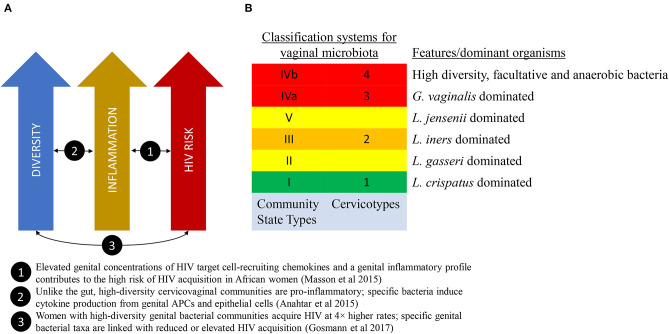
The link between vaginal microbiome composition and HIV acquisition risk. **(A)** A model that proposes that high diversity vaginal microbiota leads to localized inflammation and increased HIV acquisition risk. **(B)** Two classification schema to describe diversity of the vaginal microbiota.

There are multiple potential mechanisms that may link vaginal microbiota to poor health outcomes. An emerging theory from the HIV field proposes that high diversity non-*Lactobacillus*-dominant vaginal microbial communities, even in the absence of other sexually-transmitted infections, may promote localized inflammation and increase recruitment of activated target CD4^+^ T cells into the vaginal mucosa, thereby increasing the risk of HIV transmission per sexual exposure (Figure 1A) (Passmore et al., 2016). Evidence in support of this theory is growing (Anahtar et al., 2015; Masson et al., 2015; Passmore et al., 2016; Gosmann et al., 2017), with the implication that therapeutic modulation of vaginal microbes and/or the resultant mucosal inflammation may represent emerging targets to modulate HIV acquisition risk.

Efforts to modulate the vaginal microbiota using antibiotic or probiotic therapy have shown limited lasting or reliable success. Designing effective interventions to alter the vaginal microbiota (VMB) will require improved understanding of how the vaginal microbial communities are established and maintained across the individual lifespan and within human populations.

The focus of this paper is to review the literature about the determinants of the vaginal microbiota composition that increases risk of the inflammatory dysbiotic state and its associated adverse health outcomes. We review the existing literature on determinants of VMB composition and discuss how molecular approaches can be harnessed to advance our understanding of individual and population-level VMB composition patterns.

## What Is “Normal” ?

The link between microbial communities in the vagina and symptomatic inflammation has long been recognized. See the Historical Perspective Box 1 for an overview of clinical, pathological and molecular characterization of vaginal microbiota composition since the early 1900s.

Box 1 **Historical Perspective.**

Much of the research done on vaginal dysbiosis—i.e., an imbalance or maladaptation of the bacterial communities—was performed prior to the advent of molecular technologies, and scientists' characterization of these microbial communities relied on microscopy, culture, and clinical features. Up until the 1950s, a vaginal discharge associated with decreased *Lactobacillus* abundance was termed non-specific vaginitis. In 1955, Gardner and Duke isolated a bacterium from women with non-specific vaginitis, and this organism was later named *Gardnerella vaginalis*. With developments in microscopy and culture techniques, it became possible to distinguish between vaginal discharge caused by the parasite *Trichomonas vaginalis*, the fungus *Candida albicans* and the mixed organism condition termed bacterial vaginosis. Bacterial vaginosis was diagnosed clinically according to the Amsel criteria, or microscopically according to Nugent Score.

Roughly, symptomatic bacterial vaginosis as per Amsel criteria is a subset of all bacterial vaginosis (which includes asymptomatic cases) as per Nugent score, which in turn is a subset of vaginal dysbiosis as per molecular characterization. Because of the overlap this review considers evidence related to both bacterial vaginosis and vaginal dysbiosis.

A landmark study published in 2011 by Ravel et al. characterized the vaginal microbial communities of a cohort of healthy reproductive-age women in the United States using molecular sequencing technology (Ravel et al., 2011). These microbial communities clustered into five ‘community state types' (CST), four of which were *Lactobacillus*-dominated (Figure 1B).

Growing evidence suggests that low diversity, *Lactobacillus*-dominated VMB is associated with lower inflammation and higher diversity VMB is associated with higher inflammation, specifically activated mucosal CD4^+^ T cells (Anahtar et al., 2015). Evidence suggests that low levels of inflammation in the low diversity, *Lactobacillus*-dominated VMB is protective, and non-*Lactobacillus*-dominated higher diversity VMB (sometimes termed “dysbiotic”) is associated with risk of infections (including HIV) and obstetric complications (Anahtar et al., 2018). Notwithstanding any potential benefits of high VMB diversity which may have not yet been identified, it has been proposed that a *Lactobacillus*-dominated VMB be understood as optimal (Anahtar et al., 2018).

This low inflammation *Lactobacillus*-dominated VMB appears to be the most frequent community state in some populations—particularly Europeans and White Americans—however there is considerable heterogeneity among geographic and socio-demographic sub-populations, raising important questions about whether there is actually is a single “normal” vaginal microbiome. Many studies have shown that *Lactobacillus-*dominance is differentially present according to racial group/ethnicity, and this effect appears to be robust to adjustment for certain confounding known risk factors for bacterial vaginosis (BV), including history of STIs, douching practices and lower socioeconomic status (Ness et al., 2003). However, other potential confounding factors that may differ between population groups and influence vaginal and other microbiota, including detailed dietary, environmental and demographic have not been fully taken into consideration in these studies to date. Studies in Sub-Saharan African populations found *Lactobacillus*-dominance in <40% of asymptomatic women (Jespers et al., 2015; Gosmann et al., 2017), inspiring controversy around whether *Lactobacillus*-dominance should be considered “normal” in all populations (Ma et al., 2012; Anahtar et al., 2018).

Further complexity is introduced by the fact that an individual's VMB composition is not necessarily stable over time. An early study found that oral, gut and skin microbial communities were distinct between body sites and individuals, but an individual's microbiota showed marked variability over months, weeks, and days (Caporaso et al., 2011). Looking at the vaginal niche specifically, another study followed a cohort of 32 women over 16 weeks and sequenced their VMB at twice-weekly intervals (Gajer et al., 2012). Some patterns emerged. *L. crispatus* dominated communities were the most stable. *L. iners* dominated communities were less stable and switched into dysbiotic states more readily. Dysbiotic communities usually remained dysbiotic, but occasionally became dominated by *L. iners*. Results from longitudinal studies suggest a dynamic stability (Srinivasan et al., 2010; Santiago et al., 2011; Gajer et al., 2012; Ravel et al., 2013); where most women either maintain their CST, or oscillate between particular CSTs with some predictability. These oscillations often correspond to menses, and occasionally sexual activity. Although the relationship is not entirely clear, risk of poor reproductive or infectious outcomes may correlate to proportion of time spent in a dysbiotic state, or in terms of a tendency toward stable dysbiosis.

## Profiling The Vaginal Microbiota

Modern molecular methods have opened a range of new possibilities for the characterization of the VMB, allowing us to not only establish which microorganisms are present, but also to begin to understand their functional properties.

### 16s rRNA Taxonomic Profiling

One of the earliest molecular methods for measuring and describing VMB composition is 16s rRNA taxonomic profiling. The combination of conserved and hypervariable regions in the 16s rRNA gene makes it an ideal bacterial “barcode” and allows a microbiome sample to be efficiently characterized up to a species resolution. Using clustering methods to categorize the taxonomic profiles, we are able to associate microbiota composition with variables of interest. These community state types (Ravel et al., 2011) or cervicotypes (Anahtar et al., 2015) (see Figure 1B), have been associated with race/ethnicity, menstrual cycle (Gajer et al., 2012; Hickey et al., 2013), and inflammatory profiles (Anahtar et al., 2015; Gosmann et al., 2017).

### Metagenomic Sequencing and Strain-Level Profiling

In contrast to targeted 16s rRNA profiling, unbiased metagenomic sequencing of microbiota samples enables strain-level profiling of the microorganisms present. Strain-level information is necessary to investigate transmission patterns. Yassour et al. used this strategy to infer vertical transmission of *Bifidobacteria* species (Yassour et al., 2018). In a Fijian cohort, Brito et al. used strain-level data in the form of single nucleotide polymorphisms and flexible genomic regions to infer transmission patterns of oral- and gut-associated bacteria in the context of interpersonal relationships (Brito et al., 2019).

### Functional Genomics

Going a step further, we can characterize the VMB according to function rather than taxonomy. Metatranscriptomic, metaproteomic, and metabolomic analyses may provide more relevant data to inform causal relationships, but our knowledge is limited. We have a poor understanding of, and ability to accurately measure, metabolic products relevant to the optimal and dysbiotic VMB including short chain fatty acids and lactic acid (Aldunate et al., 2015). Metaproteomic analysis of mucosal inflammation and target cell activation in a cohort of African women has shown that minority microorganisms may have disproportionate metabolic activity and functional impact (Alisoltani et al., 2020). However, at this stage reference database limitations undermine the reliability of metaproteomics approaches.

### Culture-Based Profiling

Omics techniques provide insights into the composition and function of microbiota, but cannot fully replace conventionally cultured cells. Srinivasan et al. cultivated a set of BV-associated bacteria and then identified them using molecular methods (Srinivasan et al., 2016). Although technically complicated by the anaerobic nature of many organisms in the vaginal niche, cultured organisms may be subjected to whole genome sequencing and be used to study *in vitro* interactions to clarify causal and mechanistic relationships.

## Determinants of Vaginal Microbiota Composition

VMB composition can be understood as a result of the complex interplay between the effects of the host on the vaginal microenvironment and the effects of its microbial residents on each other. Understanding the mechanisms of host-related and microorganism-related factors is essential for effective intervention. Data on the determinants of VMB composition are limited and do not yet allow clear conclusions.

### Transmission of specific microorganisms

From a microbiological perspective, each microorganism has certain qualities and effects on the vaginal microbiota. *Lactobacillus crispatus* appears to inhibit dysbiosis (Gajer et al., 2012; Jespers et al., 2015), while *Lactobacillus iners* does not (Gajer et al., 2012; van Houdt et al., 2018). *Gardnerella vaginalis* and *Prevotella* species are often present in low abundance in healthy women and in high abundance in women with BV (Gajer et al., 2012; Wijgert et al., 2014). *G. vaginalis* has been found to exist in two distinct forms, dispersed and cohesive, with the latter being associated with biofilm formation (Swidsinski et al., 2010; Verstraelen and Swidsinski, 2013) and persistent BV. It is possible that strain-level distinctions modulate the effect of individual microbial species on the VMB which would make transmission patterns an important determinant.

#### Maternal Inoculum

There is good evidence to suggest that the infant microbiota is populated with maternal bacteria at birth, from the vagina in cases of natural delivery and from the skin in cesarean section births (Dominguez-Bello et al., 2010; Mueller et al., 2015). At birth the bacterial communities across the infant's skin, oral cavity, nasopharynx, and gut are largely undifferentiated, which suggests that a female infant's vaginal niche would also be populated with similar communities (Dominguez-Bello et al., 2010). Whether these early bacteria are still responsible for populating the vaginal niche in later life remains unclear. Phylogenetic analysis of all published *Bifidobacterium* strains suggests that vaginal and gut-associated *Bifidobacteria* are not distinct communities (Freitas and Hill, 2018) and could therefore be transmitted from the maternal vagina to the infant gastrointestinal tract. Strain-level profiling of maternal and infant microbiota has made it possible to investigate transmission patterns directly.

Yassour et al. looked at vertical transmission of gut bacteria in 44 mother-infant pairs (Yassour et al., 2018) and found matching strains of *Bifidobacteria* and *Bacteroides* spp. in most cases. Notably, the mother's dominant strain was often transmitted, and her secondary strain occasionally.

In a study primarily looking at the microbiota of premenarchal girls (Hickey et al., 2015), Hickey et al. performed a secondary analysis looking for similarities between the VMB of girls and their mothers. There were no general trends, and the data was not powered for significance but there were some notable similarities observed. Some pairs shared the same dominant taxa (including *G. vaginalis, L. iners, L. crispatus*, and *Bifidobacterium*) over multiple visits. Identifying true biological patterns between mother-daughter pairs could have important implications for understanding the genetic and environmental influences on microbiome composition.

#### Sexual Transmission

It has long been argued that BV is akin to a sexually transmitted condition (Schwebke and Desmond, 2005), based on a strongly consistent epidemiological profile (Fethers et al., 2008). Both are associated with early sexual debut, recent intercourse, multiple partners and unprotected sex. Herpes Simples Virus 2 is an important risk factor for bacterial vaginosis and is sexually transmitted (Esber et al., 2015). Furthermore, the high concordance of BV status in women who have sex with women suggests sexual transmission (Marrazzo et al., 2010; Vodstrcil et al., 2015). Studies have found various characteristics of male sexual partners to be associated with BV, which also suggests transmission. In a US study, race of male sexual partner was strongly associated with risk for BV. White women with African American male partners had double the risk of those with Caucasian male partners (Klebanoff et al., 2010). Similarly, in pregnancy, an African American father was associated with increased risk of BV as compared to a father of another race (Simhan et al., 2008). Male circumcision reduces the risk of BV in female partners, which suggests sexual transmission (Gray et al., 2009).

Sexual activity has been shown to influence VMB composition in a cohort of young, relatively sexually inexperienced women (Vodstrcil et al., 2017). While the VMB is largely resilient to sex-induced changes, penile-vaginal sex was found to increase *G. vaginalis* clade diversity, suggesting sexual transmission of commensal and potentially pathogenic clades. Taken together the data suggest that BV is a sexually transmitted infection but leave unanswered the question of whether sexual transmission influences the composition of the VMB more broadly. There have been cases where a change in composition was associated with sexual activity (Gajer et al., 2012), but it remains to be seen whether this result will be replicated in the future high resolution longitudinal data or whether the finding that the VMB of most women are resilient to sex-induced changes will be more widely confirmed.

#### Non-sexual Transmission

One study comparing the microbial communities of dwellings and their human inhabitants found that microbiota differed substantially between homes, that each home's microbiota was identifiable by family and that humans were the probable vectors (Lax et al., 2014). Another study investigating the microbial communities found in dust collecting in dormitory airvents showed that using bacterial genera alone one could predict the sex of room occupants with 79% accuracy (Luongo et al., 2017), and that taxa associated with the vaginal microbiome were more common in female occupied rooms and skin and male urogenital taxa with male-occupied rooms. These findings suggest a significant degree of dispersion of vaginal microorganisms within a living space and could allow for microbial transmission via direct contact.

It has been found that cohabiting family members share a common microbiota among them and with their pets (Song et al., 2013). Song et al. compared the skin, oral and gut microbiota of 60 families (including 159 human and 36 dogs). In particular, skin microbiota is most similar for cohabiting human-human and human-pet pairs, which is consistent with transmission by touch. Gut microbial patterns cluster by age, while skin and oral compositions do not. There is very little data on how household co-residence may influence composition of the VMB.

Extrapolating from this, one could conclude that vaginal microorganisms would be primarily transmitted via sexual contact, but consideration of the range of clothing, bathing, and other non-sexual objects that may come into contact with the human vagina does not simplify matters.

### Host Factors

#### Genetics

One possible mechanism for host-related effects is the innate individual immunological and metabolic characteristics conferred by host genetics. A re-analysis of Human Microbiome Project (HMP) data showed an association between key genes involved in immune function and certain bacterial abundances in four body niches, not including the vagina. Leptin signaling genes were highly correlated with microbiome composition, as well as several immunity-related pathways, including Melatonin Signaling, JAK/Stat Signaling, Chemokine Signaling, CXCR4 Signaling, and Role of Pattern Recognition Receptors in Recognition of Bacteria and Viruses (Blekhman et al., 2015). A more recent study by Kolde et al.—also a secondary analysis of HMP data—found greater microbial community similarity between first-degree relatives, as suggested by marker genes, and in the absence of collected familial relationship metadata (Kolde et al., 2018). This study included vaginal datasets but the number of first-degree relatives identified left them underpowered to reach any niche-specific conclusions. The host genetics dimension could also account for observed race and ethnicity differences in VMB patterns. It is important to note that these findings are easily confounded by shared environment or other sociodemographic factors. No work has yet been done to directly link host genetics to VMB composition.

#### Physiology

Composition dynamics of the VMB are significantly influenced by female reproductive physiology and changing levels of female sex hormones.

##### Menarche

Very few studies have longitudinally characterized the vaginal microbiome pre- and post-menarche of young women prior to sexual debut, thereby directly addressing the question of the impact of menarche on vaginal microbiome. Thoma et al. characterized changes to the vaginal microbiota and pH over time among sexually inactive adolescents in Rakai, Uganda using Nugent Gram-stain (Thoma et al., 2011a). During the 2-year period of follow-up they found that premenarchal girls (who never experienced menarche during the period of follow-up) had initially low counts of large Gram-positive rods (likely *Lactobacillus* spp.) that increased as they grew older. These changes correlated with a drop in vaginal pH. Interestingly, those participants who experienced menarche during the study period or who where menarchal throughout the study period showed no significant change in VMB composition during the 2-year study period. These results suggest that the anaerobic predominant VMB of childhood (Hill et al., 1995) may become *Lactobacillus*-dominated under the influence of rising levels of estrogen rather than as a direct result of menarche. Confirming this idea, Hickey et al. found that *Lactobacillus* spp. dominated the microbiota of pre-adolescent women and that the VMB of adolescents was similar to those of reproductive-age women (Hickey et al., 2015). In a study of 13–18 year old menarchal adolescents, Yamamoto et al. found compositional and structural similarity between the vaginal microbiota of menarcheal adolescents and adults (Yamamoto et al., 2009).

##### Menses

Song et al. longitudinally characterized the vaginal microbiota of healthy young women (Song et al., 2020) and showed that vaginal microbial diversity increased and *Lactobacillus* abundance decreased during menses. The use of hormonal contraceptives disrupted these cyclical changes, suggesting that that temporal variation correlates with the known effects of estradiol on VMB composition. Interestingly, however, multiple studies have shown that these cyclical fluctuations in community state type over the menstrual cycle are not present in all menstruating women, even in those who are not using hormonal contraceptives. Gajer et al. longitudinally followed 32 reproductive age women (Gajer et al., 2012) and found that some women demonstrated cyclical alterations in vaginal community that correlated to menstrual cycle, while others had stable community states that showed no alteration over time.

##### Pregnancy

Romero et al. longitudinally followed non-pregnant women and women who were pregnant and delivered without complication at term (Romero et al., 2014). 16S rRNA gene sequencing showed higher abundance of *Lactobacillus* spp. (with the exception of *L. iners*) in pregnant women compared to non-pregnant women. In contrast high complexity bacterial community states with anaerobic dominance were less frequent in pregnant women. Longitudinal analysis revealed that the vaginal microbiota was more stable over time in pregnant compared to non-pregnant women.

##### Menopause

Hillier et al. found that *Lactobacilli* were detected at lower frequency and coliform Gram-negative organisms were detected at higher frequency in post-menopausal women (Hillier and Lau, 1997). Similarly, Pabich et al. found that postmenopausal women have lower levels of vaginal *Lactobacilli* and increased vaginal *E. coli* compared to pre-menopausal women (Pabich et al., 2003). Estrogen replacement therapy has been shown to restore vaginal *Lactobacilli*, confirming the strong relationship between estradiol and *Lactobacillus* spp. (Muhleisen and Herbst-Kralovetz, 2016).

#### Behavior

It is likely that host behaviors influence the vaginal microenvironment and its hospitability for some microbial residents.

Given the role of estrogen which is thought to mediate the stabilizing effect of pregnancy on VMB composition (Romero et al., 2014) and cyclical instability induced by the menstrual cycle (Gajer et al., 2012; Hickey et al., 2013), it is likely that hormonal contraception usage would be an important influence. However, systematic review and meta-analyses reveal inconsistent results thus far, largely due to the variety of hormonal contraceptive methods available (Achilles and Hillier, 2013; Vodstrcil et al., 2013; Anahtar et al., 2018). Vaginal douching has been associated with increased risk of incident BV (Lewis et al., 2017), but not VMB composition explicitly, and is unlikely to be a long-lasting determinant.

Although *S. aureus* colonization is associated with tampon use (Jacquemond et al., 2018), it has been difficult to tease out menses-related from menstrual hygiene-related alterations to the overall vaginal microbiota. A study that compared the menstrual fluid microbiome from tampons cultured *S. aureus* from the menstrual blood of 40% of healthy women but found no clear difference in the other microbiota between those who had *S. aureus* colonization and those who did not. In a small longitudinal study comparing mid-cycle and during menses vaginal microbiota of women with baseline *Lactobacillus*-dominant VMB who used study-provided menstrual hygiene products, Hickey et al. found that menses itself caused alterations of the microbiota from baseline (Hickey et al., 2013), but that there were not specific patterns associated with particular products. Differences in menstrual hygiene product use between geographic and demographic sub-populations (Romo and Berenson, 2012) may be a confounding factor in studies that show differences between in the VMB composition between racial or ethnic groups.

Dietary patterns have been consistently associated with gut microbial composition and function (Louis et al., 2007; Tilg, 2010; Edwards, 2017), but there is less evidence for its effect on the VMB. One study in non-pregnant women found increased dietary fat intake to be associated with increased risk of BV and severe BV, whereas increased intake of folate, vitamin A, and calcium is associated with decreased risk of severe BV (Neggers et al., 2007). Another looked at dietary indices, and found that both glycaemic load and nutrient density were associated with greater BV prevalence and glycaemic load was associated with an increase in BV persistence and acquisition (Thoma et al., 2011b). Unfortunately, diet is a complex psychosocial behavior and these findings could easily be confounded by socioeconomic factors.

The association between smoking and vaginal dysbiosis is very well-established (Cherpes et al., 2008; Brotman et al., 2014; Fettweis et al., 2014). Antibiotic use is known to disrupt eubiosis in multiple body niches, but a number of studies in the vagina specifically found that these disruptions do not appear to last (Ding and Schloss, 2014; Dunlop et al., 2019). A number of studies have identified chronic psychosocial stress as an important mediator of vaginal health (Holtgrave and Crosby, 2003; Nansel et al., 2006), as mediated by the effect of cortisol on glycogen deposition and mucosal immune function (Amabebe and Anumba, 2018). Chronically elevated cortisol levels are known to be immunosuppressive and deplete glycogen stores, potentially impacting the VMB because glycogen-rich epithelium encourages *Lactobacillus* growth. This relationship between stress and dysbiosis offers a potential mechanism for the prevalence of dysbiosis in racial and ethnic minority groups worldwide (Kenyon et al., 2013; Lewis et al., 2017) and offers, in addition to the other potential social confounders, a possible explanation to the widely observed “racial” differences in VMB composition.

## From Association to Causation in The Vaginal Microbiome

The technical advances made possible by high-throughput sequencing and other functional omics approaches, offer potential to advance our understanding of the determinants of VMB composition. Advances in human subject designs and statistical measurements of association or causation are also required. Most published studies that attempt to relate VMB to adverse health outcomes are limited to cross-sectional data that allow identification of associations between potentially related variables. Intervention studies are the most rigorous way to establish causal relationships, and trials of probiotic therapy have commenced and are beginning to show promising results for the treatment of BV (Cohen et al., 2020). Interventional studies using probiotics to modulate risk of adverse health outcomes via manipulation of the VMB are planned. While awaiting results of these experimental intervention studies, there are a range of analytical approaches from which we can infer causal relationships.

Longitudinal data can suggest causation. It is through associating longitudinal data with temporally associated metadata that it was possible to identify the relationship between community state type and menstruation (Gajer et al., 2012; Ravel et al., 2013).

Probabilistic models offer a method to infer causal relationships from complex and disparate datasets. Noyes et al. used Bayesian Networks to explore the associations between sexual and hygiene behaviors and VMB composition (Noyes et al., 2018). They identified both novel and known relationships; including *L. iners* with older age, *Ureaplasma* with previous pap smear, and *L. crispatus* with any contraceptive use. More recent Bayesian Network algorithms are optimized for incomplete and uncertain datasets, and these may be particularly well-suited for identifying relevant determinants. It is important to note that any associations identified in this manner still require further investigation of their validity and significance.

It is possible to go a step further toward identifying causal patterns from observational data, using causal calculus, or *do*-calculus (Pearl and Bareinboim, 2014), a causal reasoning system based on Bayesian Networks. This approach requires a hypothesized causal structure which is then tested against the available data, in the form of a probability distribution. With our expanding datasets, this approach may yield important insights into the mechanistic pathways in the vaginal and other microbiota.

## Conclusion

The link between non-*Lactobacillus*-dominated high diversity VMB and a variety of adverse outcomes for women's sexual and reproductive health has been well-established. This suggests that treatment or prevention of vaginal dysbiosis may improve women's health outcomes. Attempts at intervention have thus far shown limited success. We will need a clearer understanding of what determines vaginal microbiota compositions in order to effectively modulate them. An important barrier is our lack of understanding of the best way to measure the optimal VMB, be it compositional or functional, whether reliable biomarkers exist, and which measurements may be translatable for clinical utility.

Work has been done to investigate determinants of microbial composition patterns in other body niches, but very little in the female genital tract so far. While the evidence reviewed in this manuscript suggests hypotheses, the current evidence does not allow any niche-specific conclusions about the determinants of vaginal dysbiosis. Other limitations of the currently available evidence include a mismatch between molecular VMB characterization and comprehensive epidemiological metadata, and a focus on STI clinics and young Caucasian women.

There is an urgent need to better understand VMB composition patterns, across the lifespan, outside of STI clinics and in sub-Saharan Africa where the risk of HIV-acquisition remains a public health emergency. More work is needed to clarify relationships between clinical symptoms, host genetic, host behavior, and molecular VMB profiles. These insights will lay the groundwork for novel and targeted interventional approaches to alleviate the morbidity and mortality associated with vaginal dysbiosis.

## Author Contributions

YM, DK, TO, and EW contributed to conception and design of this review. YM performed the original search and wrote the first draft of the manuscript. DK, TO, and EW substantially edited and reorganized sections of the manuscript. All authors contributed to manuscript revision and have read and approved the submitted version.

## Conflict of Interest

The authors declare that the research was conducted in the absence of any commercial or financial relationships that could be construed as a potential conflict of interest.
